# Earth Dreams: Reimagining ARPA for Health of People, Places and Planet

**DOI:** 10.3390/ijerph182312788

**Published:** 2021-12-03

**Authors:** Alan C. Logan, Brian M. Berman, Susan L. Prescott

**Affiliations:** 1 Nova Institute for Health of People, Places and Planet, 1407 Fleet Street, Baltimore, MD 21231, USA; alanxlogan@gmail.com (A.C.L.); bberman@tiih.org (B.M.B.); 2 Center for Integrative Medicine, Department of Family and Community Medicine, University of Maryland School of Medicine, Baltimore, MD 21201, USA; 3 inVIVO Planetary Health, Worldwide Universities Network (WUN), Baltimore, MD 21231, USA; 4 ORIGINS Project, Telethon Kids Institute, Perth Children’s Hospital, University of Western Australia, 15 Hospital Avenue, Nedlands, WE 6009, Australia

**Keywords:** ARPA-H, COVID-19, non-communicable diseases (NCDs), mental health crisis, value systems, health inequities, environmental degradation, planetary health, social justice, social and economic determinants of health, biodiversity losses, climate change, developmental origins of health and disease (DOHaD), the exposome

## Abstract

Bold new approaches are urgently needed to overcome global health challenges. The proposed Advanced Research Projects Agency for Health (ARPA-H) is intended to provide rapid health breakthroughs. While new technologies for earlier disease detection and more effective treatment are critical, we urge equal attention be given to the wider (physical, emotional, social, political, and economic) environmental ecosystems driving the non-communicable disease (NCD) crisis in the first place. This requires an integrated, cross-sectoral vision that spans the interwoven connections affecting health across the scales of people, places, and planet. This wider “exposome” perspective considers biopsychosocial factors that promote resilience and reduce vulnerabilities of individuals and communities over time—the many variables driving health disparities. Since life course health is strongly determined by early life environments, early interventions should be prioritized as a matter of effectiveness and social justice. Here, we explore the origins of the Advanced Research Project Agency and point to its potential to build integrated solutions, with wisdom and ethical value systems as a compass. Since the planned ARPA-H is anticipated to spawn international collaborations, the imagined concept is of relevance to a broad audience of researchers. With appropriate input, the quest for health equity through personalized, precision medicine while deconstructing unacceptable structural inequities may be accelerated.

## 1. Introduction

**Figure 1:**
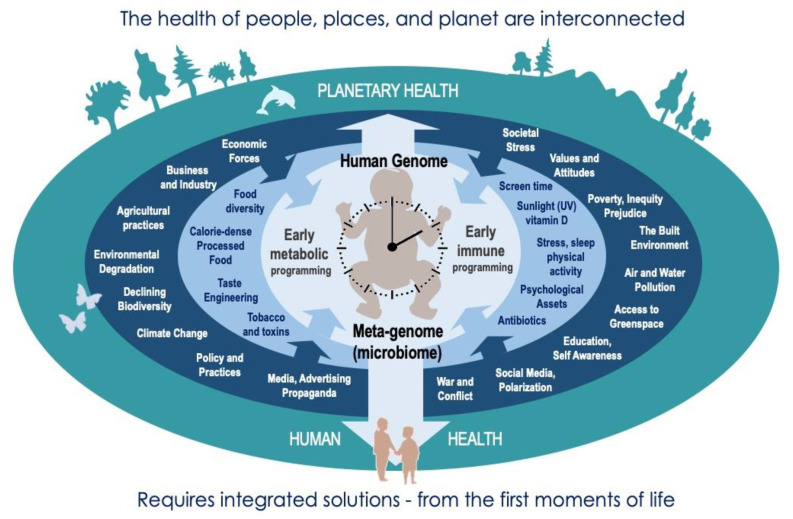
The need for an integrated “exposome” approach to health on all scales: that recognises and addresses the interconnected factors that promote resilience and decrease vulnerability from the first moments of life—including the attitudes and value systems that govern these—for mutual benefits to individuals, communities, and natural systems upon which we depend.

Increasing human disease, distress, and despair are inextricably bound with degradation and destruction of environments at all scales, and the underlying social, economic, and political value systems [1,2,3,4]. Understanding these intricate connections and erasing artificial lines between the biological, psychological, social, and cultural aspects of health in the modern environment is essential to improving the health of people, places, and planet. Indeed, the chronic pandemic of non-communicable diseases (NCDs) remains greatest threat to human health (including COVID-19 vulnerability) and cannot be solved without addressing these wider determinants of health and health behaviors. Emerging concepts of planetary health have greatly increased awareness of the ways in which the health of individuals, communities, and the Earth’s natural systems are interdependent [1,2,3,4] (Figure 1)—emphasizing the need for multilateral strategies through deep structural change. No matter how innovative, a “downstream” targeted focus on disease will ultimately fail if not considered in tandem with the “upstream” total lived experience, which determines vulnerabilities of individuals and communities over time—calling for a far more comprehensive “exposome” approach that balances reductionism with holism [5]. It equally requires that these efforts are applied with greater conscience and moral wisdom, placing greater value on equitable, mutualistic, ecological solutions [6].

In this context we explore the pending establishment of the Advanced Research Projects Agency for Health (ARPA-H). With bipartisan support, including that of United States President Joseph R. Biden, the draft legislation proposes more than USD 6.5 billion to operate the new agency. Accord to this legislation, the mission of ARPA-H is to “speed” transformational innovation and application and implementation of “health breakthroughs”. The agency will “tackle bold challenges” and “support high-risk exploration that could establish entirely new paradigms” [7]. ARPA-H has received much attention in high-impact journals such as *Science*, the *Lancet*, and *Nature* [8,9,10], as discussed below. However, these discussions have largely focused on “biomedicine” breakthroughs—pharmaceuticals and high-technology—despite the described mission of ARPA-H as requiring a far more comprehensive approach.

**Figure 2:**
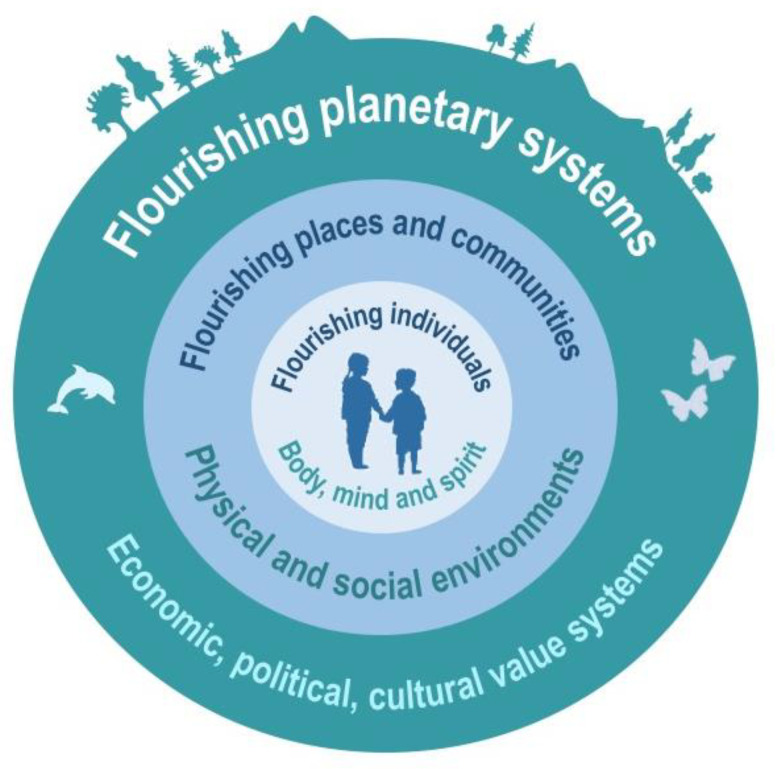
A laudable goal—flourishing as more than the absence of disease: equitable flourishing and fulfillment of individuals requires societies, systems and values that promote mutual flourishing. It also depends on overcoming the systemic factors that undermine this, recognizing the interconnected ways these influence the wellbeing of people, places, and planet.

Our discussion below takes the position that *health* (as it pertains to the “H” in the ARPA-H acronym) should be viewed as far more than the absence of disease—but rather the presence of flourishing, and the capacity to reach our fullest potential [11,12]. This includes physical and mental health, character strengths, meaning and purpose, and the financial and material resources linked to sustained flourishing over time [11,12] (Figure 2). First, we trace the origins of ARPA in the Department of Defense, and why its management operations are often promoted for use in non-military sectors. Next, we underscore the need for a more comprehensive approach to health goals, which elucidates ways in which multiple dimensions of the wider exposome (physical, emotional, social, political, and economic “ecosystems”) influence individual biology. Notably, we will explore how advances in “omics” technologies, including microbiome science, have revealed the intricate connections between personal biology and the exposome—as the total lived experience over time. This also serves as a cautionary tale for ARPA-H, one that shows how an exclusive focus on drugs, apps and tests can obscure the interconnections and complexity of NCD prevention and treatment. Finally, we set forth an “upstream” exposome approach that could help ensure ARPA-H fulfills its intended mission. Although ARPA-H is an American agency, if it develops in a similar way to ARPA, it will be of direct and indirect relevance to international research collaborators, scientists and scholars, especially those concerned with environmental and public health. Already, European researchers are underscoring that ARPA-H will cultivate partnerships and cooperation with European and global stakeholders [13].

## 2. Origins of ARPA—Space Dreams

In December 1957 a small byline in many US newspapers noted the Pentagon’s plan to establish the Advanced Research Projects Agency (ARPA). Proponents of ARPA argued that the need for an agency where ideas considered “too fantastic for immediate research” could be financed and pursued in channels that did not compete with practical research needs [14]. A place for big dreams “*to direct study and eventual creation of such things as space platforms and weapons still undreamed of*” [15]. By early January, 1958, the press announced that “*one of earliest efforts of the ARPA probably will be to perfect a television-equipped, unmanned satellite to serve as a vehicle for reconnaissance over literally the whole world*” [16]. ARPA became official on 7 February 1958—its new director, Roy W. Johnson, dubbed the “Space Czar” [17].

History would show that ARPA (by the mid-1970s referred to as DARPA, with “D” for Department of Defense—we will use the ARPA acronym for convenience) did far more than perfect reconnaissance satellites, build the undreamt-of weapons and space platforms. The many “side-benefits” of ARPA-supported research projects include the development of the Internet (via ARPA-NET), GPS, voice recognition, phase I trials of mRNA vaccines, drones, and untold amounts of technology used by individuals all over the planet [18,19]. By many measures, ARPA is an American success story, and investments in many so-called fantastical ideas have paid off handsomely. The extent to which ARPA’s military investments (including those the public knows little about) have potentially compromised public health and the environment remains a matter of debate. When ARPA is presented in glowing terms, a certain romanticization is inevitable, eliding, for example, that ARPA’s herbicidal defoliant (Agent Orange via “Project AGILE”) is now held responsible for acute and long-term health catastrophes in Vietnam and beyond [20,21].

## 3. Processes That Promote Quantum Advances and Circumvent Bureaucracy

Notwithstanding that an almost exclusive focus on its historical successes may obscure less healthy aspects of ARPA investments, it is the managerial structure and methods of operation that have made ARPA a model potentially worth cloning. From its inception, ARPA began as a place with little bureaucracy. It did not conduct original research as an agency. Rather, its program managers held enormous sway in deciding which projects were worthy of pursuit. Essentially, program managers only had to convince two individuals of the merits of a proposal, their own office director and the ARPA director [22]. These were typically individuals “on loan” for two to five years from an academic or private-sector outfit. For new initiatives, this underscores the need for guiding values and principles that equitably safeguard the health and future of people, places, and planet [6]—in an era when these are under far greater threat than ever before.

In the pursuit of innovation and technological advances, the ARPA model was subsequently adopted by several nations. In 2019, Germany launched the Federal Agency for Disruptive Innovation (SPRIN-D) with a budget of around EUR 1 billion (USD 1.2 billion) over 10 years, and in 2020, the United Kingdom announced plans to launch the Advanced Research and Invention Agency (ARIA), which will receive GBP 800 million (USD 1.1 billion) to cover its first few years. ARIA will be led by “visionary researchers” who will “identify and back the most ambitious, cutting-edge areas of research and technology” [23]. Importantly, the international ARPA-style efforts are now being directed at health-related social problems that otherwise seem insurmountable. For example, in 2018 the Japanese government launched the ARPA-inspired Moonshot Research and Development Program, which “aims to create disruptive innovations from Japan and promotes challenging R&D based on revolutionary concepts that are not simply the extension of existing technologies, i.e., moonshots.” It is guided by two principles: “Set ambitious goals and concepts to attract people for social issues that are difficult to tackle but will have profound impact once resolved,” and “aim to achieve the Moonshot Goals by bringing together wisdom from all over the world under the direction of top researchers.” The initial budget of Moonshot is JPY 100 billion (USD 911 million) over five years, with seven goals set out: i. Overcoming limitations of body, brain, space and time; ii. Ultra-early disease prediction and intervention; iii. Coevolution of AI and robots; iv. Sustainable resource circulation to recover the global environment; v. Sustainable global food supply; vi. Fault-tolerant universal quantum computer; vii. Sustainable care systems for enjoying one’s life until 100 years old [24].

## 4. Making the Case for an ARPA for Health

In 1996, physician Robert Cook-Deegan argued that the United States National Institutes of Health (NIH) needed an ARPA of its own. While Cook-Deegan applauded the success and importance of NIH peer-review processes in funding decisions, he underscored classic examples of innovators denied funding through the bureaucratically steeped NIH peer-review system. He made the larger point that the NIH peer-review process generally squeeze out novelty in favor of “safe” projects that are less likely to result in quantum change. He proposed that creating an ARPA at NIH—in parallel to ongoing “tried and true” peer review processes—was more likely to disrupt the slow pace of change, and lead to remarkable health breakthroughs [25]. This approach is also better suited for emerging or cross-sectoral approaches without a natural disciplinary base, or a field unlikely to be developed by ongoing industrial or academic efforts.

## 5. Becoming a Reality—Earth Dreams

Twenty-five years after Cook-Deegan’s proposal, ARPA-H may soon be a reality. In June, 2021, United States Congressional Representatives Diana DeGette (D-CO) and Fred Upton (R-MI) released draft legislation that is intended to create an Advanced Research Projects Agency for Health (ARPA-H). Supported by President Joseph R. Biden, the draft legislation authorizes more than USD 6.5 billion to operate the new agency. There has been debate as to whether ARPA-H should be situated within NIH—opponents suggest that contagion from NIH bureaucracy and its incremental rigidity will find a way to undermine the bold, risk-taking projects [26]. The House Appropriations Committee has recommended that ARPA-H sit within the NIH, but its physical offices should be outside of NIH’s main campus in Bethesda, MD [27].

With little opposition and bipartisan support, ARPA-H may be operational as early as 2022. Already, NIH leaders have outlined their own vision of ARPA-H in the pages of *Science* [8]. The mission of ARPA-H, according to the group, could be to make pivotal investments in “*…solutions that have the potential to transform important areas of medicine and health for the benefit of all patients and that cannot be readily accomplished through traditional research and commercial activity*”. The group further states that the focus of ARPA-H should be broad, ranging from the molecular to the societal.

This is laudable and very encouraging for those involved in public health and environmental research. Even more promising, the NIH group acknowledged that ARPA-H cannot be simply “cloned” from the original Department of Defense ARPA because the products of the latter (e.g., military hardware) are far *less* complex than the human behavior and social factors that determine health. The authors also mention that equity considerations—including race, ethnicity, gender/gender identity, sexual orientation, income, sexual orientation, and disability—should be woven into the ARPA-H mission.

## 6. The Risk of Limiting the Scope and Impact of ARPA-H before it Begins

So influential is the *Science* editorial, that the Biden Administration has copied the text and placed it on the White House website [28]. However, in the shaded box titled “Examples of Potential Projects” the NIH group cited “wearable devices”, “apps” and “platforms” to better deliver pharmaceuticals for non-communicable disease (NCD) management—specifically, hypertension and diabetes.

While this is laudable, at no point does the NIH group suggest that ARPA-H might go “upstream” and create breakthroughs that leverage immense volumes of research on the factors that propel NCDs and worsen communicable disease outcomes (such as COVID-19 severity and mortality [29,30,31]). This is an urgent priority, as Americans who have consistently lived in poor neighborhoods are 46% more likely to be obese, and 52% more likely to have hypertension compared to peers without a life in neighborhood poverty [32].

If ARPA-H is to fulfill its mission, it will require equal attention to the wider (physical, emotional, social, political, and economic) ecosystems driving the non-communicable disease (NCD) crisis and decreasing US life expectancy [33,34] in the first place. While corporate innovation helped ARPA’s defense mission, as it likely will do with ARPA-H, it is important to remember that corporate operations, through the heavily marketed spread of unhealthy products into disadvantaged communities, have been central to the NCD crisis in North America and globally [35,36,37,38,39]. Food insecurity increases the likelihood that ultra-processed foods will be consumed [40]. There is a strong case that targeted marketing is an important component of structural poverty and racism [41,42]. These conflicts must be addressed, ideally through promoting corporate responsibility towards structural shifts, as a fundamental part of the solutions.

There also needs to be far greater focus on mental health. While the *Science* ARPA-H editorial made liberal reference to cancer, blood sugar, diabetes, blood pressure, hypertension and infections, reference to “mental health”, “depression” or “anxiety” were notably absent. Mental disorders are both upstream and downstream of “physical” illness—diagnosable depression and anxiety—and subclinical and subthreshold variants that may otherwise escape detection [43]—are linked to subsequent cardiovascular disease (CVD) and other NCDs and are also noted sequalae of most NCDs [44]. Indeed, comorbidity research shows 60% of patients with anxiety or major depressive disorder have one or more chronic NCD [45]. Thus, concepts of “no health without mental health” should sit at the heart of the mission of ARPA-H.

In his own remarks, President Biden has stated that ARPA-H should be set up not only to detect and treat, but to find breakthroughs that “prevent” chronic diseases [46]. No matter how innovative it might be, a downstream targeted focus on disease will ultimately fail if not considered in tandem with the upstream total lived experience (the exposome, discussed below), which determines vulnerabilities of individuals and communities over time—understanding the value and responsibility of acting early in life when both physiological responses, attitudes and behaviors are established and most vulnerable to adversity [47,48]. We suggest that it is past time for scholarly debate concerning the scope of ARPA-H; as *Health* is the goal, we might start by going upstream to the very definition of health, and ask “whose health, what health?”

## 7. ARPA-H and the Exposome

Just months before the 1969 moon landing, microbiologist and environmentalist Rene Dubos (1901–1982) called for a “*massive effort similar to the one initiated by the National Aeronautics and Space Administration (NASA)*” that would to help medical scientists understand health—factors that promote or detract from health over the life course— from the perspective of the total environment [49]. The idea was to move beyond single-variable bench science (as important as that is) and study “genes x the total environment” over time, with exposures that also include social policies and practices [50,51]. It was becoming clear that biological responses of the human organism are a product of accumulated experiences and may differ across time depending on shifting environmental variables.

Today, the study of the total accumulated environmental exposures (both detrimental and beneficial) that can help predict the biological responses of the “total organism to the total environment” *over time* is referred to as “exposome” science [47]. Exposome research has been made possible by the new era of “omics” technologies—that is, the ability to simultaneously measure large numbers of biomolecules representing functional proteins (proteomics), metabolites (metabolomics), gene expression (epigenomics and transcriptomics), and genetic influences on drug/isolated nutrient metabolism (pharmacogenomics) [52]. These markers can help illuminate the biological implications of the total lived experience of individuals and entire populations [53,54]. When combined at larger scales, these measurements can be analyzed by experts in bioinformatics and biostatistics to predict personalized biological responses—while potentially enhancing large-scale clinical care in profound ways [55].

Returning to the example of marketing inexpensive and heavily marketed ultra-processed and fast-food to disadvantaged communities, this had been shown to translate into an absence of serum antioxidants in already vulnerable populations [56,57]. This commercially driven consumption of non-nutritive ingredients in tandem with an absence of antioxidants represents overlapping, but apparently distinct, risk factor for NCDs [58,59,60,61].

Of particular importance to the exposome is emerging microbiome science—the study of microbes and their functional genetic material operating within a specific ecological niche such as gut, skin and other anatomical locations [62]. This research demonstrates that otherwise unseen microbes are not merely a surrogate marker of the total lived experience, they are actively transducing the total environment (e.g., social advantage or disadvantage, “lifestyle” and its neoliberal marketing drivers) into biological responses, potentially influencing behavior and promoting or hindering flourishing [63,64].

## 8. An Example and a Cautionary Tale

In the early 2000s, it was postulated that the administration of non-pathogenic microbes (e.g., probiotics) could influence neurocognition and mental health [65,66]. This seemed outlandish at the time. However, less than a decade later, millions of dollars were being poured into the search for microbiome-manipulating “biomedicines” in the realm of neuropsychiatry, and headlines such as “drugging the microbiome” were appearing in *Nature* and other journals [67]. Without question, the microbiome is justifiably the subject of much excitement—research shows changes in the gut microbiota (vs. controls) in many mental disorders [68], volumes of preclinical studies have provided mechanisms by which microbes can influence the nervous system (e.g., directly via the vagus nerve and indirectly through the immune system), and small-scale clinical trials show benefit of select microbial strains in depression, anxiety and other mental disorders [69].

Yet, almost twenty years on, and with untold amounts of money spent, the concept of “drugging the microbiome”, at least as far as mental health or precision medicine goes, has not advanced to a point that even closely matches the hype [70,71,72]. Similar hype has plagued the human genome-precision medicine field [73]; genome-wide association studies have succeeded in demonstrating that very few chronic diseases are mediated by genes alone. Moreover, creating a distinction between groups of patients based on select biomarkers can leave large groups (so-called “molecularly unstratified” patients) without the “hope” of a particular intervention, and interfere with providing equitable access to care for all patients [74].

## 9. New Perspectives on the Ecology of Social Disadvantage

Much like the observation that NCDs are often linked to socioeconomic position—such that NCDs are disproportionally shouldered by the disadvantaged—research has shown that the composition and diversity of gut (and oral) microbes are similarly associated with socioeconomic position in various populations [75,76,77,78,79]. It is also known that functional changes to the microbiome are mediated by many “lifestyle” factors, including stress [80,81,82], sleep [83], exercise [84], tobacco use [85], and, of course, short and long-term dietary choices [86,87,88].

For example, biological responses to a single meal or short-term dietary intervention are mediated by the microbiome, which is itself is a product of the person’s lived experience, including the background diet [89,90,91,92,93]. Select gut microbial species are associated with healthy dietary habits and, if viewed separately, overlap with species associated with favorable cardiometabolic and postprandial markers, even in individuals without clinically manifested disease [94]. Since the gut microbiome modulates local and systemic immune function, and has been implicated in most NCDs, it should be little surprise that gut (as well as oral, and lung) microbiota are emerging as a factor in determining COVID-19 susceptibility, severity and survivability [95,96,97]. Furthermore, the efficacy of biological treatments, including breakthrough drug candidates, the sort imagined by ARPA-H, may also be determined by the very microbiome that is shaped by the lived experience [98,99,100,101].

Background diet is considered an independent variable, but how independent is it, really? Stress and sleep restriction, both tied to microbiome alterations, are provocateurs of unhealthy dietary choices. What about systemic poverty and racism? When researchers merely provoke laboratory perceptions of poverty, powerlessness or the perception of belonging to an under-privileged “out-group”—let alone actually being in poverty or a minority group—the result is altered dietary choices in the direction of low-nutrient, high-calorie foods [102,103]. It can also produce taste-based perceptual shifts that increase sensitivity to the presence of energy in beverages [104]. Again, that is a result of briefly invoked perceptions in a lab, an “in situ” from which the research subject can readily escape; the reality of everyday discrimination can manifest as inflammation and metabolic dysfunction [105,106,107], and, as mentioned above, compound the inequity of the total environment, nutritional and otherwise.

Examining separate lines of research, we can see that maternal smoking can influence select gut microbes in children, microbes that are, in turn, associated with childhood cognition [108]. Physical activity appears to alter microbiota in subjects with obesity, microbes that might otherwise influence aspects of metabolism [109,110]. Consider also that gut bacterial diversity and richness has been shown to decrease when depressive symptoms increase, and that lower gut bacterial diversity and richness may provide a path to later health threats [111]. At virtually every practical and theoretical turn, the promotors of dysbiosis as shown in human and/or experimental research—environmental pollutants, crowding, acoustic stress, heat stress, circadian disruptions—are burdens shouldered by socioeconomically disadvantaged individuals and communities [63].

Taken as a whole, the emerging microbiome science allows scientists to visualize how individual and community disadvantage “gets under the skin” and into the mind–-body interface [112,113]. Thus, from the ARPA-H perspective, it should encourage influential program managers to examine the structural forces that drive “inequities” of the biologically relevant microbiome. Many of the factors that drive dysbiosis are considered “lifestyle” choices that can be remedied by simple individual interventions, whereas they are really a downstream consequence of a wholly unjust environment, one that is engineered to dysbiosis by default [64].

## 10. Asking the Right Questions

“*It’s the questions that we need to discover, because the answers preexist. If we ask the right questions, the answers will come*”

Jonas Salk, 1972 [114].

As ARPA-H begins its journey toward discovery, we encourage careful selection of program managers who are capable of asking the right questions—this means program managers with a deep understanding of the science of structural poverty and racism, and the commercial determinants of health. Salk, the discoverer of the polio vaccine, held that in order to address complex health issues, including those related to the larger environment, collaboration by scientists and scholars from multiple sectors was essential to “asking the right questions”, so that the correct paths of query might be set forth. ARPA-H program managers should be drawn not only from experts in biomedicine and basic science but also from experts in environmental research, behavioral medicine and public health. The program managers should understand how exposome science, as discussed below, can unmask the drivers of disease and dis-ease, so that broad, long-lasting solutions can be discovered. In our view this will increase the odds that the mission of ARPA-H—to prevent disease and tackle bold challenges—will prioritize whole person health, and health for all.

Socioeconomic disadvantage is accompanied by higher levels of chronic psychosocial stress and daily hassles [115]. Put simply, the stress of structural poverty is bad for health [116]. In the case of minority ethnic and racial groups, the effects are compounded by everyday racism. This refers to micro, meso and macro scale racial/ethnic based discrimination activated by unequal power structures (and social relations) resulting in the unequal treatment and access to resources or services [117,118]; from the exposome perspective, lower socioeconomic position and neighborhood-level deprivation have been associated with significantly higher biomarkers of metabolic dysregulation, inflammation and oxidative stress [119,120,121,122,123,124,125,126,127]. Repetitive stimulation of compensatory physiological responses (e.g., immune, cardiovascular, neuroendocrine) can lead to metabolic dysregulation and cellular damage, which in turn can influence behavior and disease. The collective toll of this physiological wear and tear is known as allostatic load [128].

Exposome science emphasizes that certain windows of vulnerability (for disease risk) and opportunity (for health promotion) are especially important [129]. As mentioned above, in the context of ARPA-H, this means that socioeconomic advantage or disadvantage can produce differing biological responses to specific “beneficial” or “detrimental” exposures. For example, spending time in safe, high quality green space or consuming a calorie-dense, nutritionally poor fast-food meal will produce biological outcomes that depend on many other background variables [48,130].

An exposome approach presents an opportunity to query the specifics of health inequities, such as structural racism. For example, among the biomarkers of cellular age, telomere length may provide insight; at the individual level and over the long-term, shorter telomere length (indicating long-term cellular damage) has been noted in association with racial discrimination [131,132], and neighborhood disadvantage measured via census tract adult education, poverty rate, and unemployment, are associated with shorter telomere length [133] At the larger scale, examinations of telomere length and neighborhood characteristics are providing a better understanding of the ways in which exposure to neighborhood level (historical and current) discrimination can influence cellular damage [134,135].

Critically, this calls for an integrated, cross-sectoral vision that spans the interwoven connections affecting health across the scales of people, places, and planet. Initiatives like ARPA-H are uniquely placed to build this broader exposome perspective, which will not only enhance the quest for health equity through personalized, precision medicine but encourage cross-sectoral partnerships—as human health ultimately depends on addressing our many environmental challenges.

In a *Nature* book review of Sharon Weinberger’s critical examination of ARPA in 2017 (*The Imagineers of War* [136]), MIT history of science professor David Kaiser wondered how ARPA would fare in the context of the contemporary crisis of misinformation—its greatest challenge now is that global communications networks are accelerating the pace with which “*basic elements of reality now routinely dismissed as partisan talking points*” [137]. Thus, any vision of ARPA-H must include an investment in undoing the “infodemic”, and at the same time, acknowledging the limitations of biotechnology that merely papers over the structural forces that drive inequity [138]. It is wonderful that ARPA made early investments in mRNA vaccines, but that will only get us so far if vast segments of the public refuse to take such vaccines because of what they read on ARPA’s other invention—the Internet.

## 11. Conclusions

“*We must try to imagine the kinds of surroundings and ways of life we desire, lest we end up with a jumble of technologies and counter-technologies that will eventually smother body and soul.*”

Rene Dubos, 1971 [139].

Privileging technology as the sole pathway to personal and planetary wellness, rather than an understanding of how technology influences humankind’s relationship to each other and the natural world, was a primary concern of Dubos. When the National Institutes of Health began to study health disparities in earnest, in the early 2000s, the initial focus was almost exclusively on the biological differences that might separate groups. While that was important, the National Institute on Minority Health and Health Disparities has since helped to shift the scientific community’s focus on non-biological factors such as socioeconomics, politics, discrimination, culture, and environment in relation to health disparities. Our concern is that ARPA-H might begin its journey by privileging technology in a way that elides the same non-biological factors that underpin the necessary health breakthroughs and, at the same time, by not asking the right questions, require the need for yet further technologies to undo the unintended consequences of how technology might be utilized.

There has been much excitement surrounding genome-wide association studies, yet exposome science has helped to eliminate genes as a factor in why NCDs are shouldered by disadvantaged populations, and why NCDs are increasing over time and in non-random ways [47]. Bold new approaches are urgently needed to overcome the mounting health crisis. The proposed Advanced Research Projects Agency for Health (ARPA-H) situated within the National Institutes of Health provides a promising opportunity to achieve this, through a vision that (1) takes an integrated approach that considers entire ecosystems and all populations, with vulnerable groups in mind; (2) is based on wisdom as morally grounded excellence [6]; and (3) promotes whole person health and holistic care throughout communities [140,141].

While new technologies for earlier detection and more effective treatment are critical, we urge equal attention to the wider (physical, emotional, social, political, and economic) ecosystems driving the non-communicable disease (NCD) crisis in the first place. No matter how innovative, a “downstream” targeted focus on disease will ultimately fail if not considered in tandem with the “upstream” total lived experience (the exposome), which determines vulnerabilities of individuals and communities over time. The exposome holds promise to blend holism and reductionism, and it improve the integration of biological, environmental and social data—the bio-markers and socio-markers [5].

ARPA-H is set to encourage visionary or so-called “Blue Sky” thinking. The NIH group concludes its *Science* editorial by stating that “*ARPA-H can help shape the future of health and medicine by transforming the seemingly impossible into reality. The time to do this is now*”. We agree. ARPA is now part of mid-century American folklore, beginning its mission with fantastical space dreams. When ARPA-H begins its mission, Earth dreams, those that envision equitable health for persons, places, and planet, should be welcome.

## Data Availability

Not applicable.
